# Strategic Design of Ethanol Oxidation Catalysts: From Active Metal Selection to Mechanistic Insights and Performance Engineering

**DOI:** 10.3390/nano15191477

**Published:** 2025-09-26

**Authors:** Di Liu, Qingqing Lv, Dahai Zheng, Chenhui Zhou, Shuchang Chen, Kaiyang Zhang, Suqin Han, Hui-Zi Huang, Yufeng Zhang, Liwei Chen

**Affiliations:** 1 Department of Pharmaceutical Engineering, School of Life and Health Sciences, Huzhou College, Huzhou 313000, China; liudi0535@163.com (D.L.); 18268384471@163.com (C.Z.); 19857299050@163.com (S.C.); 2 Beijing Advanced Innovation Center for Soft Matter Science and Engineering, Beijing University of Chemical Technology, Beijing 100029, China; 2021400329@buct.edu.cn; 3 School of Chemical Engineering & Technology, China University of Mining and Technology, Xuzhou 221116, China; 4 College of Biological and Chemical Engineering, Qilu Institute of Technology, Jinan 250200, China; zhangky05@foxmail.com (K.Z.); suqinhan@qlit.edu.cn (S.H.); 5 Ministry of Education Key Laboratory of Cluster Science, School of Chemistry and Chemical Engineering, Beijing Institute of Technology, Beijing 100081, China; 3120235806@bit.edu.cn; 6 Jiangsu Key Laboratory of Zero-Carbon Energy Development and System Integration, Nanjing Xiaozhuang University, Nanjing 211171, China

**Keywords:** ethanol oxidation reaction (EOR), electrocatalysis, anode catalysts, direct ethanol fuel cells (DEFCs), noble metals catalysts, non-noble metals catalysts

## Abstract

The ethanol oxidation reaction (EOR) is a key process for direct ethanol fuel cells (DEFCs), offering a high-energy-density and carbon-neutral pathway for sustainable energy conversion. However, the practical implementation of DEFCs is significantly hindered by the EOR due to its sluggish kinetics, complex multi-electron transfer pathways, and severe catalyst poisoning by carbonaceous intermediates. This review provides a comprehensive and mechanistically grounded overview of recent advances in EOR electrocatalysts, with a particular emphasis on the structure–activity relationships of noble metals (Pt, Pd, Rh, Au) and non-noble metals. The effects of catalyst composition, surface structure, and electronic configuration on C–C bond cleavage efficiency, product selectivity (C1 vs. C2), and CO tolerance are critically evaluated. Special attention is given to the mechanistic distinctions among different metal systems, highlighting how these factors influence reaction pathways and catalytic behavior. Key performance-enhancing strategies—including alloying, nanostructuring, surface defect engineering, and support interactions—are systematically discussed, with mechanistic insights supported by in situ characterization and theoretical modeling. Finally, this review identifies major challenges and emerging opportunities, outlining rational design principles for next-generation EOR catalysts that integrate high activity, durability, and scalability for real-world DEFC applications.

## 1. Introduction

As the global energy demand continues to rise and environmental concerns become increasingly urgent, the development of sustainable and clean energy technologies has garnered significant attention [1,2,3,4]. Among various renewable energy sources, ethanol, a widely available biofuel, has emerged as a promising alternative due to its high energy density, ease of storage, and safe transportability, particularly in comparison to hydrogen [5,6]. Direct ethanol fuel cells (DEFCs), which convert ethanol into electricity through electrochemical oxidation, offer a highly efficient, portable, and environmentally friendly energy solution [7,8]. Unlike proton exchange membrane fuel cells (PEMFCs) that rely on hydrogen and face significant challenges related to storage, distribution, and infrastructure, ethanol remains in liquid form under ambient conditions, making it a more practical and scalable energy carrier. This advantage combined with ethanol’s renewable nature and widespread availability from agricultural sources positions DEFCs as a key technology in the transition towards a low-carbon energy future [9].

The ethanol oxidation reaction (EOR) at the anode of DEFCs is a complex multi-step electrochemical process that significantly influences fuel cell efficiency [10,11]. The EOR mechanism is generally classified into two competing pathways: the C1 pathway, which involves a 12-electron transfer to completely oxidize ethanol to CO_2_ (or CO_3_^2−^ in alkaline solutions), and the C2 pathway, which primarily leads to the formation of partial oxidation products such as CH_3_COOH (or CH_3_COO^−^ in alkaline solutions) with a 4-electron transfer and/or acetaldehyde (CH_3_CHO) through a 2-electron transfer, respectively [12,13,14]. While the complete oxidation of ethanol via the C1 pathway is theoretically desirable for maximizing energy output, its realization remains challenging due to the strong adsorption of carbon monoxide (CO_ads_) on catalysts, which leads to electrode poisoning and reduced catalytic efficiency.

Despite extensive experimental and computational investigations into the EOR process, a comprehensive and consistent understanding of the EOR mechanism remains elusive. Experimental techniques, such as in situ Fourier transform infrared spectroscopy (FTIR) and online differential electrochemical mass try (DEMS), have been widely employed to identify reaction intermediates, products, and pathways [15,16]. Additionally, theoretical studies have utilized computational methods, such as density functional theory (DFT), to model reaction energetics and elucidate potential mechanisms [17,18]. However, discrepancies in reported mechanisms persist due to variations in experimental conditions, catalyst compositions, and analytical methodologies, highlighting the inherent complexity of the reaction. Overcoming these challenges requires a deeper understanding of reaction pathways, surface interactions, and kinetic limitations, which are crucial for advancing DEFC technology and improving overall energy conversion efficiency.

The performance of EOR highly depends on the design of electrocatalysts, which face major challenges in simultaneously achieving high activity and selectivity and long-term stability. While noble metals such as platinum (Pt) and palladium (Pd) exhibit excellent catalytic activity, they suffer from high cost, limited availability, and susceptibility to CO poisoning, which significantly hinders their commercial viability [19,20]. Moreover, Pt-based catalysts often demonstrate insufficient C1 selectivity, leading to the accumulation of incomplete oxidation byproducts (CH_3_CHO and CH_3_COOH), which lowers energy efficiency. Furthermore, Pt catalysts are prone to structural degradation due to particle aggregation, dissolution, or oxidation, further limiting their long-term performance. To address these limitations, various strategies have been explored, including alloying, surface modification, nanostructuring, support engineering, and electronic structure modulation, to enhance corrosion resistance and poison tolerance [12,13,21]. Given these challenges, extensive research has been devoted to developing alternative catalysts with improved performance.

Despite the substantial progress in EOR, most existing reviews have either focused on individual catalyst classes or emphasized mechanistic investigations. A critical gap persists in the form of a systematic framework that integrates diverse classes of active metal catalysts, correlating their intrinsic properties with catalytic performance. In this review, we present a comprehensive comparative analysis of recent advances in electrocatalysts for EOR, with a particular focus on systems based on Pt, Pd, Rh, Au, and non-noble metals, together with characterization techniques and evaluation methodologies that establish correlations between catalyst structure and electrochemical performance. We systematically dissect their catalytic behaviors by correlating composition, structure, and surface chemistry with intrinsic activity, selectivity, and stability. Through an integrated analysis of experimental insights and theoretical investigations, we elucidate mechanistic intricacies and identify performance-governing factors, including electronic structure modulation, interfacial engineering, and dynamic reconstruction, as well as others. In addition, we highlight key obstacles to practical application—including CO poisoning, incomplete oxidation, and catalyst degradation—and outline future research for designing next-generation EOR catalysts with optimized performance. This review is intended to serve as a guiding framework for accelerating the development of high-performance EOR catalysts and to stimulate innovation in sustainable alcohol-based energy conversion technologies.

## 2. Reaction Mechanism for EOR

**Figure 1:**
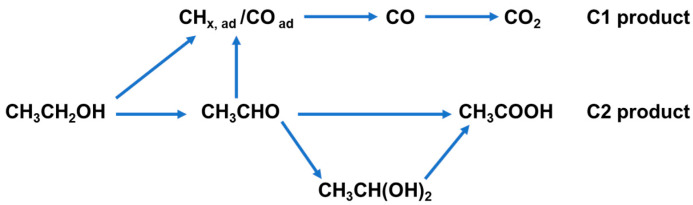
Possible reaction pathways for EOR in an acidic medium.

In acidic conditions, EOR predominantly proceeds through two competing pathways (Figure 1). The C2 pathway involves the stepwise dehydrogenation of ethanol to CH_3_CHO, followed by further oxidation to CH_3_COOH. In contrast, the C1 pathway requires C–C bond cleavage, producing adsorbed intermediates (CH_x_, CO) that can be further oxidized to CO_2_. However, the sluggish oxidation kinetics of CO intermediates often lead to severe poisoning of Pt-based catalysts, thereby reducing catalytic efficiency and stability under acidic conditions.

**Figure 2:**
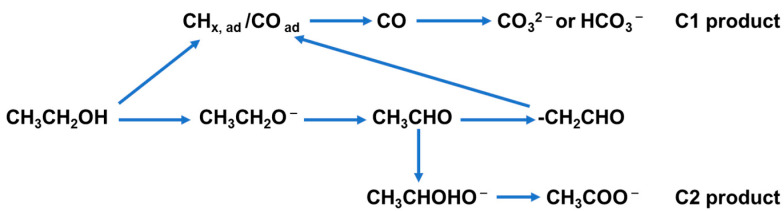
Possible reaction pathways for EOR in an alkaline medium.

In alkaline media, EOR follows similar C1 and C2 pathways, but the overall reaction kinetics are improved due to the facile adsorption and activation of OH^−^ species (Figure 2). CH_3_CHO remains the primary intermediate in the C2 pathway, which yields CH_3_COO^−^. Simultaneously, the C1 pathway is more effectively promoted because surface OH^−^ accelerates the oxidation of intermediates to CO_3_^2−^/HCO_3_^−^, thus mitigating catalyst poisoning. Compared with acidic conditions, alkaline operation not only enhances reaction rates but also favors more complete oxidation, thereby improving both catalytic activity and long-term durability.

## 3. Characterization Techniques and Evaluation Method of EOR Catalysts

### 3.1. Physicochemical Characterization

A comprehensive understanding of the physicochemical properties of EOR catalysts is essential to establish the structure–performance correlations that govern catalytic performance. Advanced characterization techniques provide critical insights into the crystal structure, morphology, surface composition, and dynamic evolution of catalysts under operating conditions.

#### 3.1.1. Structural and Morphological Analysis

Structural features such as crystal phase, lattice strain, and alloying degree can be elucidated by X-ray diffraction (XRD), which reveals the degree of crystallinity and phase composition. Transmission electron microscopy (TEM, HRTEM, and HAADF-STEM) offers high-resolution visualization of particle morphology, size distribution, and interfacial heterostructures, enabling the identification of nanoscale architectures that promote ethanol adsorption and bond activation. Scanning electron microscopy (SEM) further complements these studies by providing information on particle dispersion and electrode surface roughness, both of which affect catalytic accessibility and electrochemical performance.

#### 3.1.2. Surface Composition and Electronic States

The surface chemical environment plays a decisive role in EOR pathways. X-ray photoelectron spectroscopy (XPS) is widely employed to determine the oxidation states of surface atoms and monitor electron density shifts upon alloying or heteroatom doping. Energy-dispersive spectroscopy (EDS) allows spatially resolved elemental mapping, clarifying the distribution of active components across the catalyst matrix. Furthermore, X-ray absorption spectroscopy (XAS, including XANES and EXAFS) provides quantitative insights into coordination environments and electronic modulations, thereby establishing correlations between local atomic structure and catalytic function.

#### 3.1.3. In Situ/Operando Characterization

To capture the dynamic behavior of catalysts during ethanol oxidation, in situ and operando characterization techniques have become indispensable. In situ FTIR and Raman spectroscopy enable real-time identification of reaction intermediates, such as CO, CH_3_CHO, and CH_3_COOH, which are crucial for evaluating C–C bond cleavage efficiency and product selectivity [17]. Electrochemical mass spectrometry (EC-MS) facilitates direct monitoring of gaseous and volatile products (e.g., CO_2_ versus C_2_ species), providing quantitative evidence for pathway analysis. Surface-enhanced infrared absorption spectroscopy (SEIRAS) and surface-enhanced Raman spectroscopy (SERS) further enhance sensitivity toward low-concentration adsorbates, allowing detection of transient species under realistic electrochemical potentials [18]. These approaches collectively advance mechanistic understanding and guide the rational design of next-generation EOR catalysts.

### 3.2. Electrochemical Performance Evaluation of EOR Catalysts

Electrochemical testing is a fundamental step in assessing the activity, selectivity, and durability of EOR catalysts. While physicochemical characterization reveals structural and compositional features, electrochemical performance metrics directly determine the practical applicability of catalysts for EOR and DEFC systems. This section summarizes the methodologies and key indicators used to evaluate catalytic performance.

#### 3.2.1. Activity

Catalytic activity is typically evaluated in a three-electrode configuration. Cyclic voltammetry (CV) provides essential parameters such as the onset potential, oxidation peak potential, and current density, which together reflect the intrinsic catalytic activity and kinetics of ethanol oxidation. Linear sweep voltammetry (LSV) yields polarization curves that enable direct comparison of activity across different catalysts under identical conditions. Tafel slope analysis is employed to gain mechanistic insights by identifying the rate-determining steps, offering a deeper understanding of how catalyst composition influences electron transfer and intermediate formation. Key performance indicators include a lower onset potential, higher mass activity (mA·mg^−1^_metal_), and specific activity normalized to the electrochemically active surface area (ECSA, mA·cm^−2^_ECSA_).

#### 3.2.2. Selectivity

Since ethanol oxidation can proceed via both C1 and C2 pathways, selectivity toward complete oxidation to CO_2_ versus partial oxidation to acetaldehyde or acetic acid is critical for catalyst evaluation. In situ FTIR and Raman spectroscopy enable the identification of key intermediates (CO, CH_3_CHO, CH_3_COOH), thereby elucidating pathway preferences. DEMS allows direct, real-time detection of CO_2_ evolution, providing quantitative insights into C–C bond cleavage efficiency [17,18]. Additionally, high-performance liquid chromatography (HPLC) and nuclear magnetic resonance (NMR) spectroscopy can be employed to quantify liquid-phase products. The most relevant performance indicators include C1 versus C2 product selectivity and Faradaic efficiency associated with specific products, which collectively determine the practical energy efficiency of ethanol utilization.

#### 3.2.3. Stability

Catalyst stability is a decisive factor in determining long-term applicability in DEFCs. Several electrochemical protocols are used to probe the durability of EOR catalysts. Chronoamperometry (CA) measures current retention at a constant potential, simulating prolonged operation under fuel-cell-like conditions. Chronopotentiometry (CP) complements this approach by monitoring potential drift at a fixed current, which reflects catalyst degradation during steady-state operation. Accelerated durability tests (ADTs), typically involving repeated potential cycling within a defined potential window, are used to mimic harsh fuel cell environments and reveal degradation pathways such as nanoparticle agglomeration, dissolution, or support corrosion. Electrochemical impedance spectroscopy (EIS) is frequently employed to monitor the evolution of charge-transfer resistance, providing further insights into electrode kinetics and interfacial degradation.

Key stability indicators include the percentage of current retention after extended testing, the preservation of ECSA, and the resistance to potential-induced deactivation (e.g., CO poisoning). Establishing standardized stability evaluation protocols is essential to bridge the gap between laboratory-scale testing and real-world applications.

#### 3.2.4. MEA Testing in DEFCs

While three-electrode systems provide a well-controlled environment for probing the intrinsic properties of EOR catalysts—such as onset potential, mass activity, specific activity, and tolerance against poisoning—they deliberately avoid engineering complications inherent to fuel cells, including ethanol crossover, membrane resistance, and electrode coupling effects. This enables fundamental insights into structure–activity relationships, yet the translation of such intrinsic properties into practical device performance requires evaluation under realistic conditions.

MEA testing serves as a critical bridge between laboratory-scale studies and practical DEFC applications. In this configuration, catalysts are integrated into a membrane–electrode assembly (typically Nafion-based) and tested under operating conditions that account for ethanol transport, electrode–membrane interactions, and long-term durability [7,8]. Performance metrics include open circuit voltage (OCV), peak power density, current density at specific voltages, and voltage/current decay during extended operation.

Importantly, establishing the correlation between CV-derived activity parameters in three-electrode systems and actual MEA performance is essential for identifying descriptors that reliably predict catalyst behavior in devices. A systematic workflow—first benchmarking catalysts in three-electrode configurations to reveal intrinsic activity, followed by MEA evaluation to capture system-level effects—represents a rational pathway from mechanistic understanding to real-world deployment of EOR catalysts.

## 4. Electrocatalysts for EOR

The EOR is characterized by sluggish kinetics and a complex mechanism involving multiple surface-adsorbed intermediates and a variety of products and byproducts. A comprehensive understanding of the EOR mechanism is therefore crucial for advancing catalyst design and improving performance. By tuning the compositions, structure properties, coordination environments, and support interactions, the adsorption behavior of key intermediates can be optimized, leading to enhanced EOR activity, selectivity, and stability. The following section provides an overview of the EOR mechanisms of various metal-based catalysts, categorized by their active metal components, along with corresponding optimization strategies aimed at improving EOR performance.

### 4.1. Pt-Based Catalysts

**Figure 3:**
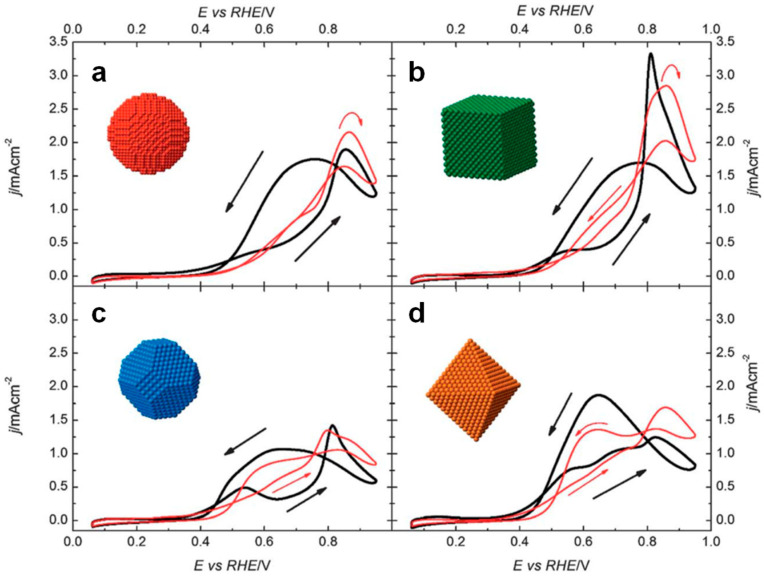
CVs of Pt nanoparticles exposed with different crystal faces in 0.5M H_2_SO_4_ + 0.2M CH_3_CH_2_OH (black line) and 0.1M HClO_4_ + 0.2 M CH_3_CH_2_OH (red line). (**a**) (poly)Pt, (**b**) (100)Pt, (**c**) (100)–(111)Pt and (**d**) (111)Pt nanoparticles. Reproduced with permission from Reference [23]. Copyright 2013, *Journal of Materials Chemistry A*.

Pt-based catalysts are the most widely utilized for EOR due to their high catalytic activity. As a surface-sensitive reaction, EOR is strongly influenced by the catalyst’s composition and surface crystal orientation [22]. IR spectra for EOR on the Pt (111) vs. Pt (100) surfaces were used to analyze CO_2_ selectivity [23]. The ration of CO_2_/acetate on Pt (100) is about 0.35, which is higher than that on Pt (111) (0.2). These data clarify that Pt-based catalysts predominantly yield C2 products with a small amount of CO_2_. Meanwhile, Pt (100) has a higher C–C scission and CO_2_ formation ability (Figure 3). These differences highlight the critical role of Pt nanoparticle morphology in determining EOR performance.

**Figure 4:**
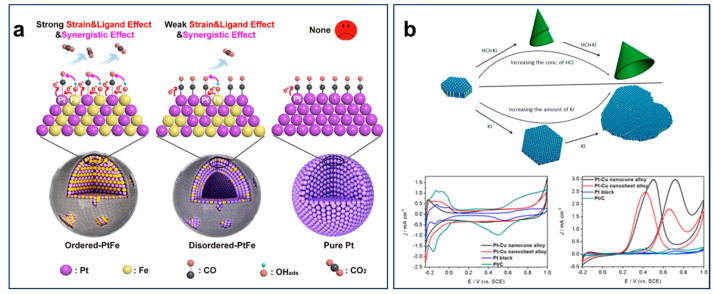
(**a**) Schematic illustration of strain effect, ligand effect, and synergistic effect for ethanol oxidation reaction on different PtFe catalysts. Reproduced with permission from Reference [29]. Copyright 2021, *Applied Surface Science*. (**b**) Scheme of Pt−Cu alloy nanocrystals and electrocatalytic properties. Reproduced with permission from Reference [31]. Copyright 2021, *Journal of American Chemical Society*.

The Pt surface is highly susceptible to poisoning by strongly adsorbed species such as CO_ads_, which are generated during the EOR process, leading to a significant reduction in both activity and stability. This issue severely limits the long-term performance and practical application of Pt-based catalysts in DEFCs. To address this challenge, alloying Pt with oxophilic metals has been widely explored as an effective strategy, as it enhances the adsorption of OH_ads_ species on the catalyst surface. The presence of OH_ads_ facilitates the oxidation of poisoning intermediates like CO_ads_, thereby improving the overall activity and durability of Pt-based catalysts [24,25,26,27,28]. Liu et al. developed a simple alloying–ordering strategy to convert pure Pt nanoparticles into size-controlled PtFe ordered intermetallic nanoparticles using thermal interdiffusion under a removable NaCl cover, with disordered counterparts obtained by adjusting Fe precursor content. The ordered PtFe catalyst exhibited stronger strain, ligand, and synergistic effects, resulting in superior activity, stability, and CO-poisoning resistance compared to disordered PtFe and commercial Pt/C (Figure 4a) [29]. This study demonstrated that alloying not only modifies the electronic properties of Pt but also influences its structural stability, highlighting the importance of controlled synthesis in catalyst design. Similarly, PtCu_3_ nanocage alloy catalysts demonstrated that the introduction of Cu modulates the electronic structure of surface Pt through alloying effects and enhances CO poisoning resistance, thereby improving catalytic activity [30]. Wang et al. successfully prepared Pt−Cu alloy nanosheets with tunable lateral sizes and thicknesses, further transforming them into nanocones via a rolling process, a technique previously limited to layered materials. These nanostructures exhibited remarkable EOR performance at room temperature in a N_2_-purged 0.5 M H_2_SO_4_ solution. The peak current density of ethanol oxidation in the positive potential scan of these nanocones is 2.97 mA cm^−2^, which is almost 22 times that of Pt black and 14 times that of Pt/C (Figure 4b) [31]. PtCo alloys have been shown to enhance ethanol adsorption through electronic modification of Pt, thereby improving EOR activity compared with monometallic Pt. The introduction of Co alters the electronic density of Pt sites, which not only weakens CO adsorption but also promotes the intermediates’ oxidation [32,33,34]. The drastic enhancement in performance emphasizes the crucial role of nanostructuring and alloying with oxophilic metals in optimizing catalyst efficiency [35]. Building on this concept, the incorporation of Bi, which exhibited strong oxygen affinity, further improves catalyst performance by facilitating OH_ads_ adsorption. The PtBi@Pt core–shell catalyst demonstrated exceptionally high EOR mass activity (9.01 A mg_pt_^−1^) and excellent stability [36]. In situ FTIR and DFT calculations revealed that the incorporation of the oxygen-affinitive Bi metal enhanced the adsorption of OH_ads_ on the PtBi@Pt surface, while also promoting CO_ads_ oxidation on neighboring Pt sites, thereby improving both the catalytic activity and stability for the EOR. The enhanced electrocatalytic activity of Pt-based alloys can be attributed to two key effects. The first is the bifunctional catalysis mechanism, in which the alloying component facilitates OH_ads_ adsorption, accelerating faster oxidation of CO_ads_ chemisorbed on adjacent Pt atoms. The second is the electronic effect, where the electronic interaction between Pt and the alloying metals alters the binding energy of adsorbed species on the catalyst surface, thereby optimizing catalytic performance.

**Figure 5:**
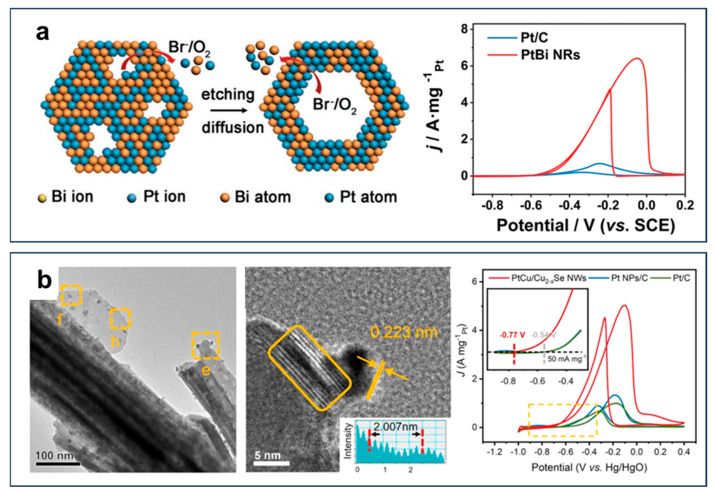
(**a**) Schematic illustration of strain effect, ligand effect, and synergistic effect for ethanol oxidation reaction on different PtBi catalysts. Reproduced with permission from Reference [37]. Copyright 2022, *Advanced Functional Materials*. (**b**) Scheme of Pt−Cu alloy nanocrystals and electrocatalytic properties. The yellow square region can be further amplified. Reproduced with permission from Reference [38]. Copyright 2021, *Nano Energy*.

Surface defect engineering has emerged as a powerful strategy for significantly enhancing the EOR performance of Pt-based catalysts. Structural defects, such as vacancies or steps, can optimize the electronic structure of Pt, increase active site exposure, and improve reactant adsorption, thereby boosting both activity and durability. For instance, PtBi nanorod (NR) alloy catalysts, characterized by abundant surface defects and non-uniform tensile strain, exhibited exceptional EOR activity, achieving a mass activity 8.5 times higher than that of commercial Pt/C (Figure 5a) [37]. These structural features optimize the electronic properties of Pt, enhance the exposure of active sites, and improve reactant adsorption and intermediate desorption. Such unique characteristics make PtBi NRs a promising candidate for advanced electrocatalysts in fuel cells and other energy conversion applications. The PtCu/Cu_2−x_Se nanowires with abundant surface defects achieved a remarkable mass activity of 5.03 A mg_Pt_^−1^, 5.0 times higher than that of commercial Pt/C (Figure 5b) [38]. These defects not only promoted C−C bond cleavage of ethanol molecules to improve C1 selectivity but also synergized with PtCu alloy effects to modulate electronic structures, as evidenced by the downshifted d-band center of surface Pt atoms that effectively weakened CO_ads_ adsorption strength. Further advancements were realized in ultrathin 2D PtPdCu nanosheets, where multimetallic composition and architecturally optimized surfaces created abundant active defect sites (corners, edges, and steps), while maintaining porous morphology to enhance mass transport [39]. This defect-dominated design enabled outstanding EOR performance, delivering a specific activity of 23.7 mA cm^−2^ with 86.4% current retention after 3600 s, substantially surpassing Pt/C. Collectively, defect engineering offers a universal strategy for designing advanced EOR catalysts with enhanced efficiency and stability.

In particular, PtSn alloys have been the most extensively studied among Pt-based catalysts. The addition of Sn provides a strong bifunctional effect, generating surface OH groups at lower potentials, which accelerate the oxidative removal of CO_ads_ and other strongly bound intermediates from Pt active sites [40,41]. At the same time, the electronic effect of Sn modifies the d-band structure of Pt, reducing the binding energy of poisoning carbonaceous species and facilitating ethanol activation. Beyond alloying effects, the choice of support material further amplifies the catalytic benefits of PtSn systems. The support directly influences nanoparticle dispersion, electronic structure, and long-term durability. Metal oxide supports such as SnO_2_ are particularly attractive due to their strong metal–support interactions and oxygen-containing surface groups. In Pt/SnO_2_ catalysts, SnO_2_ not only enhances the bifunctional effect by supplying OH species at lower potentials, thereby accelerating the oxidative removal of intermediates from adjacent Pt sites, but also induces electronic interactions that optimize the intermediates’ adsorption energies [42]. Composite supports such as SnO_2_/C further integrate the high conductivity of carbon with the hydroxyl-providing ability of oxides, leading to simultaneous improvements in activity and stability [43]. Collectively, these findings highlight that both alloying and rational support design are indispensable strategies to enhance EOR activity.

Although the EOR activity of Pt-based catalysts has significantly improved, their ability to cleave the C−C bond in ethanol molecules remains a major challenge. The increased current density observed during the ethanol electrooxidation process primarily results from the enhanced production of C2 products rather than complete oxidation to CO_2_. Notably, the CO_2_ selectivity of Pt-based catalysts remains critically low (0.5–7.5%), substantially below the operational requirements for commercial DEFCs [44]. As a result, optimizing the C1 selectivity by promoting efficient C−C bond scission has become a critical research focus in the development of advanced Pt-based electrocatalysts.

### 4.2. Pd-Based Catalysts

In alkaline electrolytes, Pd demonstrates significantly faster ethanol electrooxidation kinetics compared to Pt [45,46,47,48,49]. This enhanced performance is attributed to the inherently weaker adsorption affinity of Pd for reaction intermediates such as CO_ads_, effectively reducing catalyst poisoning during EORs [50,51,52]. Consequently, Pd-based catalysts exhibit superior EOR activity and long-term operational stability, as evidenced by their sustained catalytic performance under prolonged reaction conditions.

**Figure 6:**
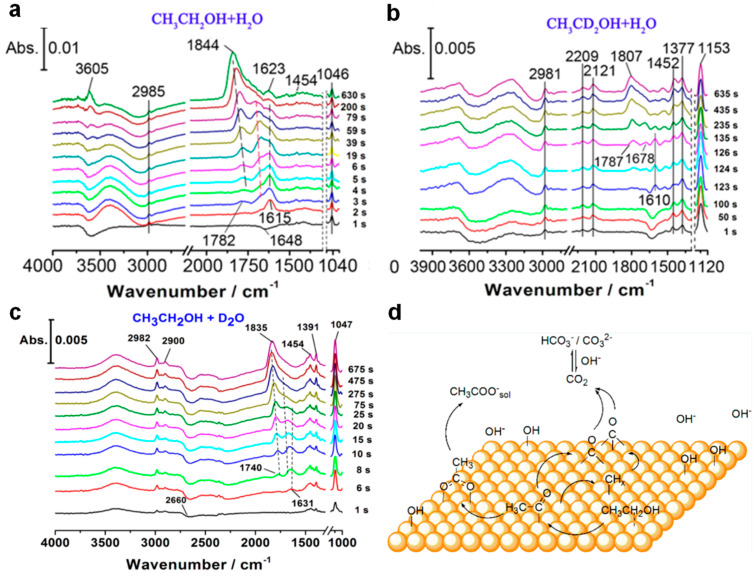
In situ FTIR of Pd electrode surface (**a**) in 0.5 M CH_3_CH_2_OH with a 0.1 M NaOH/H_2_O solution, (**b**) in 0.5 M CH_3_CD_2_OH with a 0.1 M NaOH/H_2_O solution. (**c**) Real-time ATR-SEIRA spectra on a Pd electrode in 0.5 M CH_3_CH_2_OH with a 0.1 M NaOH/D_2_O solution. The vibrational bands corresponding to interfacial CH_3_CH_2_OH or CH_3_CD_2_OH were observed at ∼2980, ∼2120, ∼1452, ∼1153, and ∼1046 cm^−1^, belong to ν(C−H) of CH_3_CH_2_OH, ν(C−D) of CH_3_CD_2_OH, δ(C−H) of CH_3_CH_2_OH, ν(C−OH) of CH_3_CD_2_OH, ν(C−OH) of CH_3_CH_2_OH. (**d**) Schematic diagram of EOR mechanism. Reproduced with permission from Reference [53]. Copyright 2014, *ACS Catalysis*.

Pd electrodes exhibited limited ethanol oxidation via the C1 pathway and primarily generated CH_3_COOH through C2 pathway. In situ FTIR coupled with H-D isotopic substitution has revealed that a minor fraction of adsorbed ethanol molecules undergoes α-C dehydrogenation to form adsorbed acetyl intermediates, followed by C−C bond cleavage into CO_ads_ and (CH_x_)_ads_ species, which subsequently react with surface-bound OH_ads_ to form CO_3_^2−^ (Figure 6) [53]. However, the majority of dehydrogenated ethanol molecules bypass C−C bond scission, with CH_3_CO_ads_ identified as the key adsorbed intermediate, resulting in CH_3_COO^−^ as the primary product under alkaline conditions. Enhancing OH_ads_ adsorption on Pd-based catalysts could facilitate the oxidative removal of CH_3_CO_ads_ species, thereby simultaneously boosting EOR activity and operational stability by mitigating intermediate accumulation.

Rational morphological and compositional engineering of Pd-based catalysts enables precise modulation of their surface geometric and electronic structures, effectively optimizing the adsorption energetics of surface intermediates and enhancing EOR performance. Pd-based alloy catalysts have emerged as a promising strategy to synergistically improve EOR activity and stability while reducing the required noble metal content, thereby lowering overall catalyst costs. Alloying Pd with transition metals (e.g., Zn [54], Cu [55], Ni [56], Fe [57], Co [58], Mn [59]) allows for multifunctional optimization by tailoring nanoscale morphology to increase active site exposure and modifying the Pd d-band electronic structure to optimize intermediate binding strength. Moreover, this strategy enhances surface OH_ads_ adsorption capacity, which is critical for improving catalytic performance. The synergistic effects facilitate rapid oxidative removal of CH_3_CO_ads_ intermediates and CO_ads_ poisoning species at adjacent Pd active sites, ultimately boosting both catalytic efficiency and long-term stability.

**Figure 7:**
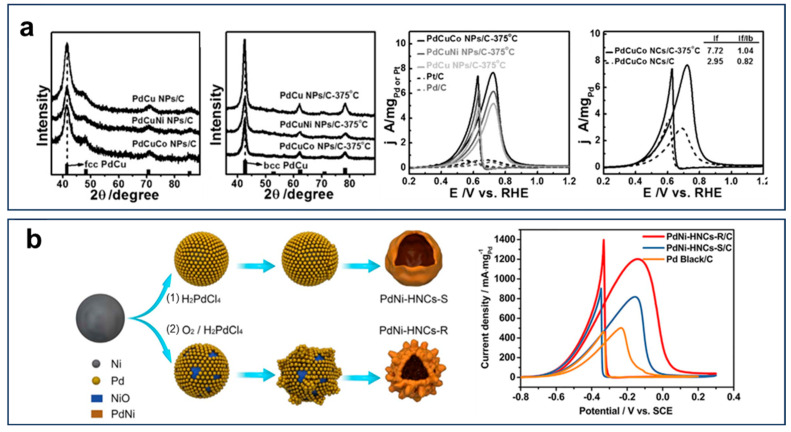
(**a**) fcc-phase XRD, bcc-phase XRD pattern, and CVs of Pd-based alloy nanoparticles. Reproduced with permission from Reference [60]. Copyright 2016, *ACS Catalysis*. (**b**) Synthesis diagram and CVs of PdNi-HNCs-R and PdNi-HNCs-S. Reproduced with permission from Reference [61]. Copyright 2017, *Nano Energy*.

Building on the strategy of alloying Pd with transition metals to optimize EOR performance, Huang et al. synthesized a series of PdCuM ternary alloy nanocatalysts, among which the PdCuCo variant exhibited the highest EOR activity, as shown in Figure 7a [60]. Theoretical calculations further revealed that the incorporation of oxyphilic metals induced synergistic ligand effects and compressive strain, collectively modulating the surface electronic structure of Pd and significantly enhancing EOR performance. Notably, the PdNi hollow nanocages (PdNi-HNCs) displayed remarkable mass activity (1201.5 mA mg_Pd_^−1^), surpassing that of commercial Pd/C (Figure 7b) [61]. Accelerated durability tests and chronoamperometric analyses confirmed the superior electrochemical stability of PdNi-HNCs. The enhancement originated from the synergistic interplay between their hierarchical hollow architecture with nanodendritic surface features, which maximized electrochemically active site accessibility, and the oxyphilic Ni-induced strengthening of OH_ads_ adsorption. The synergistic effects facilitated the rapid oxidation of CH_3_CO_ads_ intermediates at adjacent Pd sites, thereby boosting both EOR activity and stability.

**Figure 8:**
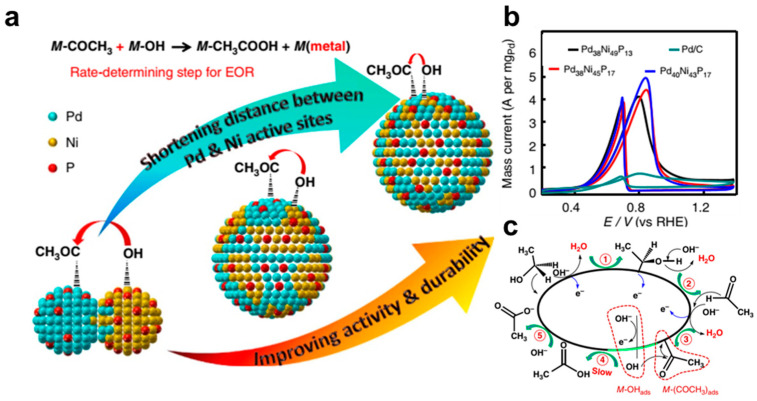
(**a**) Schematic diagram for PdNiP. (**b**) CVs of PdNiP. (**c**) EOR mechanism on PdNiP. Reproduced with permission from Reference [64]. Copyright 2022, *ACS Catalysis*.

Beyond alloying with transition metals, incorporating non-metallic elements has emerged as another effective strategy for optimizing Pd-based catalysts for EOR. Yang et al. introduced fluorine (F) atoms into Pd/N–C catalysts to modify the local coordination environment of Pd and improve both activity and durability [62]. F atoms in the Pd/N&F–C catalyst induced N atoms to migrate closer to Pd, forming Pd–N active sites. The Pd–N sites on the Pd surface not only weakened the CO adsorption but also promoted C–C bond cleavage. As a result, the Pd/N&F–C achieved a peak current density of 26.5 A mg_Pd_^−1^, with long-term stability for more than 5900 h, demonstrating great potential for practical application. Similarly, amorphous Pd-P nanoparticles demonstrated superior EOR performance compared to Pd/C [63]. The enhanced activity originated from the synergistic effects of defect-rich surfaces and the incorporation of non-metallic P, which collectively modulated the electronic structure of Pd, strengthened OH_ads_ adsorption, and facilitated ethanol electrooxidation kinetics. Further advancements were achieved with the ultrasmall (~5 nm) PdNiP nanocatalysts (Figure 8), which exhibited exceptional EOR activity, achieving a mass activity of 4.8 A mg_Pd_^−1^, which is 6.88 times higher than commercial Pd/C (0.72 A mg_Pd_^−1^) [64]. Theoretical calculations revealed that the oxyphilic Ni component significantly enriched surface OH_ads_ coverage, which synergized with adjacent Pd sites to oxidize CH_3_CO_ads_ and CO_ads_. Notably, the spatial proximity between noble metal Pd sites and oxyphilic Ni centers was identified as a key factor governing EOR efficiency, as it optimized interfacial charge transfer and intermediate oxidation dynamics. Consequently, the strategic integration of non-metallic and oxyphilic elements provided a dual-functional enhancement mechanism, simultaneously boosting EOR activity.

Despite significant advancements in enhancing the EOR activity of Pd-based catalysts in alkaline electrolytes, their limited C−C bond cleavage capability remains a major challenge. The EOR process on Pd predominantly follows the C2 pathway, which reduces the electron transfer efficiency of DEFCs and hinders their overall energy conversion performance.

### 4.3. Rh-Based Catalysts

Compared to Pt- and Pd-based catalysts, which predominantly follow ethanol partial oxidation in the C2 pathway, Rh demonstrates superior ability to cleave the C−C bond during EOR. This exceptional capability is attributed to Rh’s unique electronic structure, characterized by an optimal d-band center and a strong affinity for ethanol adsorption, which preferentially activates the C−C bond [65]. The enhanced C1 selectivity is mechanistically attributed to Rh’s ability to stabilize key reaction intermediates, such as CH_3_CO_ads_, and facilitates efficient coupling between adsorbed CO_ads_ and surface-bound OH_ads_ [66,67,68]. These intrinsic advantages position Rh-based electrocatalysts as promising candidates for DEFCs, where maximizing electron conversion efficiency through complete oxidation to CO_2_ is essential.

**Figure 9:**
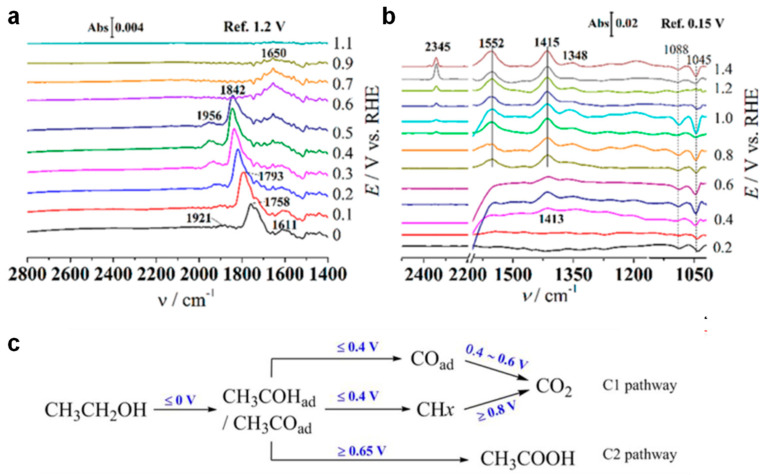
(**a**) In situ SEIRAS, (**b**) in situ FTIR, (**c**) scheme of EOR on Rh electrode. Reproduced with permission from Reference [69]. Copyright 2022, *Journal of the American Chemical Society*.

In situ FTIR was employed to investigate the changes in adsorbed species and potential reaction mechanisms during the EOR on an Rh electrode, as shown in Figure 9 [69]. At lower potentials (0.4–0.6 V vs. RHE), Rh surfaces exhibited a remarkable ability to cleave the C−C bond, with nearly 100% selectivity for the C1 pathway. However, as the potential increased (0.65–0.85 V vs. RHE), C1 selectivity decreased, while the formation of C2 products, such as CH_3_COOH, progressively increased. Notably, Rh-based catalysts exhibited a significantly lower peak potential for EOR compared to Pt- and Pd-based catalysts, highlighting their superior intrinsic activity for EOR. This potential-dependent selectivity shift was mechanistically attributed to the dynamic coverage of OH_ads_ species on Rh surfaces, which regulated the competition between complete oxidation (C1 pathway) and partial oxidation (C2 pathway) processes. These findings provide critical insights into the design of Rh-based electrocatalysts for optimizing C1 selectivity of DEFCs.

**Figure 10:**
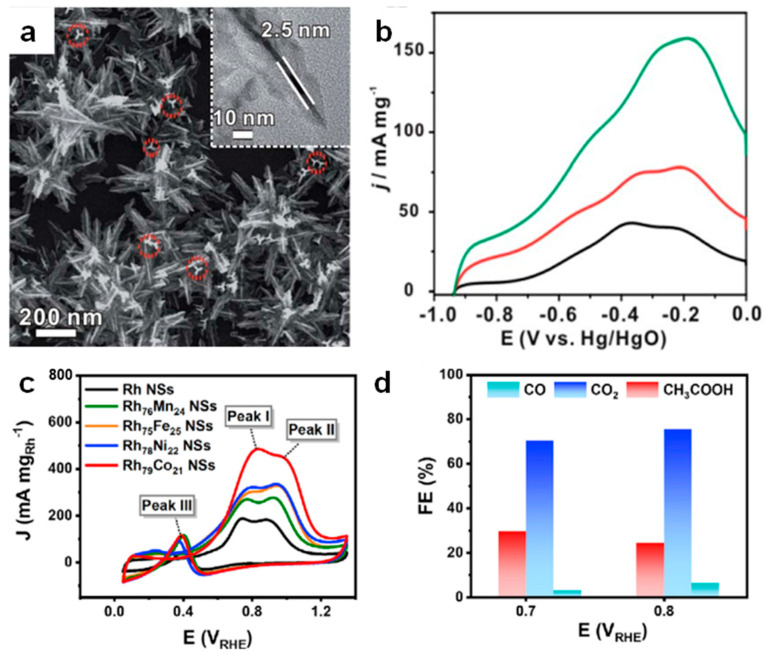
(**a**) SEM image and (**b**) polarization curve of RhNi alloy nanocrystals. Reproduced with permission from Reference [75]. Copyright 2019, *Journal of Materials Chemistry A*. (**c**) CVs and (**d**) Faraday efficiency of Rh/Rh−M nanosheet. Reproduced with permission from Reference [76]. Copyright 2022, *Chem Catalysis*.

Building on the ability of Rh to facilitate C–C bond cleavage, structural engineering has also been explored as an effective strategy to further enhance EOR activity. For instance, the hierarchically branched Rh nanostructures (CPT Rh NBs) exhibited exceptional EOR performance, achieving 2.8 A mg_Rh_^−1^ with 14.5% C1 selectivity, due to the predominance of Rh (100) facets and tensile strain effects [70]. Xie et al. developed monometallic Rh nanodendrites with multi-scale branching architectures through a seed-mediated electrochemical deposition method. The Rh nanodendrites exhibited outstanding EOR activity (3.2 A·mg_Rh_^−1^ at 0.6 V vs. RHE) with a high C_1_ selectivity of 18.2%, outperforming Rh nanoparticles [71]. Beyond structural engineering, alloying Rh with transition metals could also significantly enhance EOR activity and CO poisoning resistance [72,73,74]. Rh-Ni alloy nanodendrites exhibited enhanced EOR activity and C1 selectivity (67.2% at 0.45 V) compared to monometallic Rh and Rh black (Figure 10a,b) [75]. The oxyphilic Ni modulated Rh’s electronic structure, strengthened OH_ads_ adsorption, and accelerated CO_ads_ oxidation. Huang et al. designed ultrathin Rh/Rh-M nanosheets (M = Co, Mn, Fe, Ni), and Rh_79_Co_21_ exhibited exceptional mass activity (3.6 A·mg_Rh_^−1^) and C1 selectivity (70.3%) (Figure 10c,d) [76]. These findings highlight the synergistic effect of structural and compositional engineering in advancing Rh-based catalysts for enhanced EOR performance and durability.

**Figure 11:**
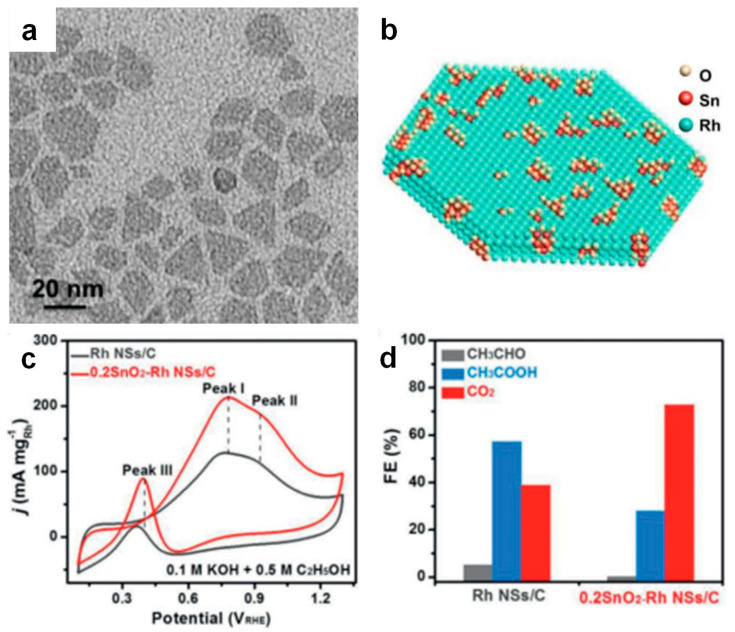
(**a**) TEM, (**b**) structure diagram, (**c**) CVs, and (**d**) Faraday efficiency of SnO_2_−Rh nanosheet. Reproduced with permission from Reference [77]. Copyright 2020, *Advanced Materials*.

The integration of Rh with non-precious metal oxides/hydroxides could effectively modulate the electronic effects of Rh-based nanomaterials and further enhance EOR performance. For instance, SnO_2_-Rh nanosheets modulated the electronic structure and could enhance ethanol and OH_ads_ adsorption, significantly improving EOR activity and C1 selectivity (Figure 11) [77]. The mass activity of SnO_2_-Rh nanosheets was 213.2 mA mg_Rh_^−1^, which was 1.7 times higher than that of Rh nanosheets. The C1 selectivity of SnO_2_-Rh nanosheets was 72.8%, notably higher than that of Rh nanosheets (38.8%). Similarly, RhPb-PbO_2_/C demonstrated outstanding EOR performance, achieving a mass activity of 2636 mA mg_Rh_^−1^, significantly outperforming commercial Rh/C and rivaling state-of-the-art Pd/C [78]. The electronic interactions between Rh and PbO_2_ effectively enhanced the adsorption of surface OH_ads_ and facilitated the removal of CO_ads_ intermediates, thereby improving both the EOR activity and C1 selectivity. The Rh-Bi(OH)_3_ further confirmed that non-precious metal oxides’ interface effects effectively promoted the cleavage of the C−C bond, achieving 81.2% C1 selectivity. The abundant OH_ads_ on the Bi surface rapidly oxidized the CO_ads_ intermediates on Rh sites, further enhancing EOR activity and stability [79]. Collectively, these studies demonstrate that the strategic combination of Rh with non-precious metal oxides/hydroxides enables synergistic electronic and interfacial engineering, significantly enhancing EOR activity, C1 selectivity, and CO tolerance in Rh-based catalysts.

**Figure 12:**
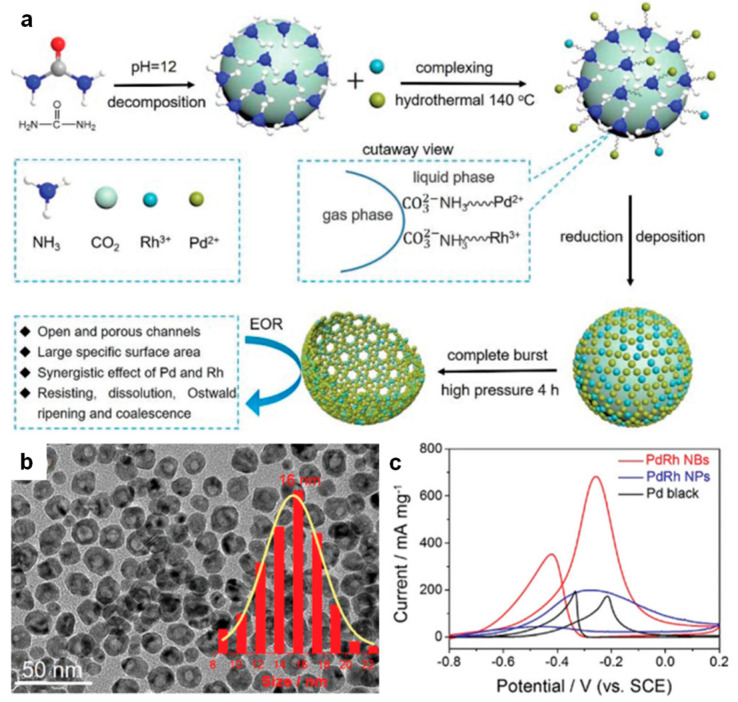
(**a**) Synthesis process, (**b**) TEM, and (**c**) CVs of PdRh NBs. Reproduced with permission from Reference [82]. Copyright 2019, *Nanoscale*.

Although the aforementioned strategies have improved the catalytic performance of Rh-based catalysts, their relatively low current density remains a significant limitation, preventing them from meeting commercial requirements. To address this challenge, integrating Rh into Pt- and Pd-based catalysts has emerged as an effective approach to enhance EOR performance by balancing high current density with improved C−C bond cleavage efficiency. The Pt_1_Rh_1_/RGO with high-density step sites was prepared by a polyol reduction method, which demonstrated a 16.2-fold increase in CO_2_ selectivity compared to commercial Pt/C [80]. Rh not only promoted C−C bond breaking ability but also strengthened OH_ads_ adsorption, enabling efficient oxidative removal of CO_ads_ intermediates from adjacent Pt sites. The PtNiRh ternary octahedrons (PtNiRh-3/C) achieved a mass activity of 2.3 A mg^−1^, which was 3.5 times higher than Pt/C, while maintaining 92% activity retention after accelerated durability testing [81]. In situ FTIR revealed that Rh downshifted the d-band center of Pt, thereby weakening CO adsorption energy and uniquely facilitating C−C cleavage. Similarly, Tang et al. designed bowl-shaped PdRh nanoarchitectures (PdRh NBs) with tunable Rh surface enrichment, which exhibited high mass activity and C1 selectivity (78%) (Figure 12) [82]. These studies collectively establish Rh as a universal promoter in Pt/Pd systems, leveraging synergistic electronic modulation, strain engineering, and bifunctional OH_ads_-mediated detoxification to enable high-performance EOR catalysts with tailored activity–selectivity profiles.

Rh-based catalysts exhibit superior C−C bond cleavage ability, leading to higher C1 selectivity compared to Pt- and Pd-based catalysts, which predominantly favor the C2 pathway. However, their relatively low current density limits their practical applications for DEFCs. To address this challenge, strategies such as structural engineering, alloying with transition metals, and integration with Pt/Pd have been employed to simultaneously enhance catalytic activity, stability, and CO tolerance.

### 4.4. Au-Based Catalysts

**Figure 13:**
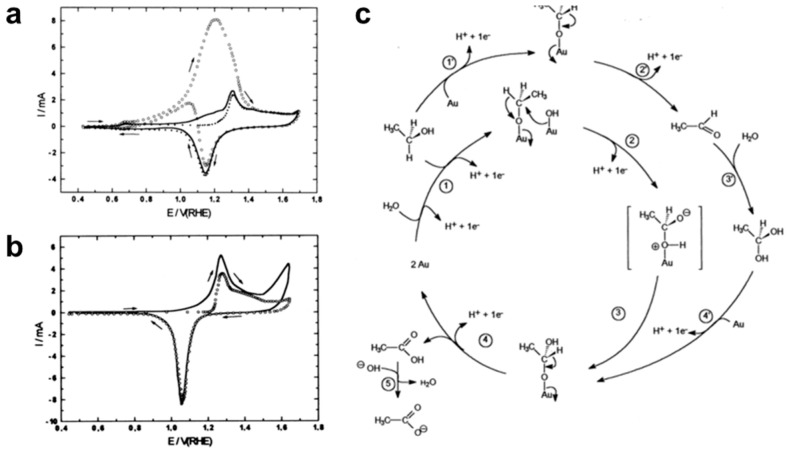
CVs on Au electrode (**a**) in 0.1 M NaOH and (**b**) 0.1 M HClO_4_. (**c**) Mechanism scheme. Steps 1–5 are the Faradaic processes. Reproduced with permission from Reference [86]. Copyright 2007, *Electrochemistry Communications*.

Although the intrinsic activity of Au is generally lower than that of Pt- and Pd-based catalysts, the weak adsorption of CO_ads_ on the Au surface minimizes CO poisoning, allowing it to maintain high activity during long-term operation [83,84]. In acidic medium, CH_3_CHO is the primary oxidation product, which undergoes further oxidation to CH_3_COOH as a secondary product with a significant induction time. This subsequent oxidation is potential-dependent, occurring at 1.2 V, as confirmed by the electrolysis of CH_3_CHO, which exclusively yields CH_3_COOH. However, at higher potential (1.7 V), CH_3_CHO remains the final product as further oxidation is negligible. Au also predominantly follows the C2 pathway under alkaline conditions, though small amounts of CO_2_ has been detected by in situ FTIR [85,86]. The EOR mechanisms on Au in acid and alkaline media are shown in Figure 13. Despite their limited intrinsic activity and C1 selectivity, Au-based catalysts exhibit excellent CO tolerance and long-term stability, making them promising candidates for future DEFC applications.

**Figure 14:**
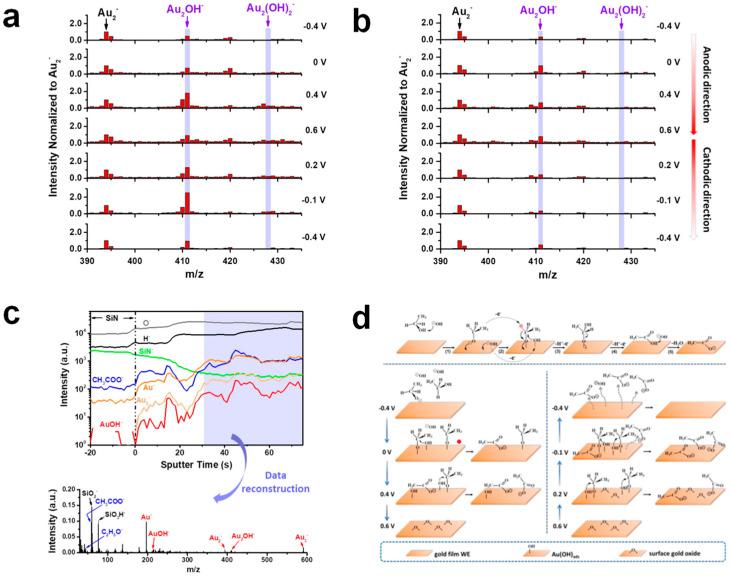
In situ mass spectrometry of surface species on Au (**a**) in 0.1 M KOH and (**b**) in 0.1 M KOH and 0.1 M ethanol. (**c**) In situ liquid-phase SIMS depth profiles on Au electrode and representative spectrum. (**d**) Reaction mechanism for the oxidation of ethanol to an acetate ion on a gold film surface in an alkaline solution. And illustration of chemical changes on the gold WE surface in 0.1 M ethanol in 0.1 M KOH solution when chosen electrode potentials were applied in the (lower left) anodic and (lower right) cathodic directions, respectively. Reproduced with permission from Reference [87]. Copyright 2019, *ACS Applied Energy Materials*.

Zhu et al. investigated the EOR process and mechanism on Au under alkaline conditions using in situ isotopic mass spectrometry (Figure 14) [87]. By employing in situ liquid-phase secondary ion mass spectrometry (SIMS) and molecular probes, they examined the surface state of Au electrodes, providing direct molecular evidence. At lower potentials, OH^−^ ions chemisorb onto the Au electrode surface, forming Au-(OH)_ads_ species, which act as the rate-determining step of the reaction. The formed Au-(OH)_ads_ species serve as an active site for ethanol molecules, facilitating oxidation through interaction with the dehydrogenated ethoxy group. As the potential increases, surface Au also undergoes oxidation, forming Au_2_O_3_, which does not adsorb OH^−^, leading to gradual deactivation. However, this oxidation process is reversible, and during the reverse scan, Au_2_O_3_ is reduced, restoring Au’s ability to adsorb OH^−^ and generating the corresponding polarization current. This study provides effective characterization evidence linking surface chemistry with catalytic performance.

**Figure 15:**
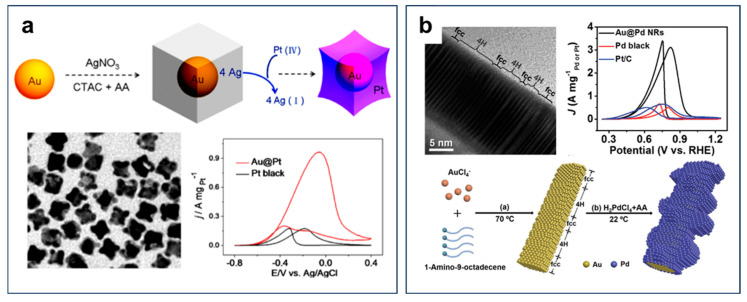
(**a**) TEM, CVs, and schematic diagram of synthesis process of PtAu@Pt NCs. Reproduced with permission from Reference [88]. Copyright 2013, *ACS Catalysis*. (**b**) TEM, CVs, and schematic diagram of synthesis process of 4H/fcc Au@Pd NRs. Reproduced with permission from Reference [89]. Copyright 2017, *Advanced Materials*.

Building on the fundamental understanding of Au-based catalysts, alloying with Pt or Pd has emerged as an effective strategy to enhance both EOR activity and stability. For instance, compared to pure Pt, PtAu@Pt NC catalysts exhibited markedly improved EOR activity and CO poisoning resistance (Figure 15a) [88]. Moreover, depositing a monolayer or submonolayer of Pd atoms on Au/C via chemical epitaxial growth resulted in the formation of a Pd_1_Au_4_ catalyst, which achieved the highest EOR activity due to the electronic interplay between Au and Pd [81]. Furthermore, Zhang et al. synthesized bimetallic core–shell 4H/fcc Au@Pd nanorods, which exhibited outstanding EOR performance (Figure 15b) [89]. Owing to the synergistic alloy effect and their unique crystalline structure, the mass activities of these 4H/fcc Au@Pd nanorods were 6.2 and 4.9 times higher than those of commercial Pd black and Pt/C, respectively. Additionally, PdNiAu_x_ ternary alloy catalysts have been shown to deliver peak power densities that are three times and two times higher than those of monometallic Pd and bimetallic PdNi catalysts, respectively [90]. The combination of Au with Pt or Pd not only enhances catalytic activity and CO tolerance but also improves resistance to long-term degradation, resulting in more durable and efficient electrocatalysts for DEFC applications.

**Figure 16:**
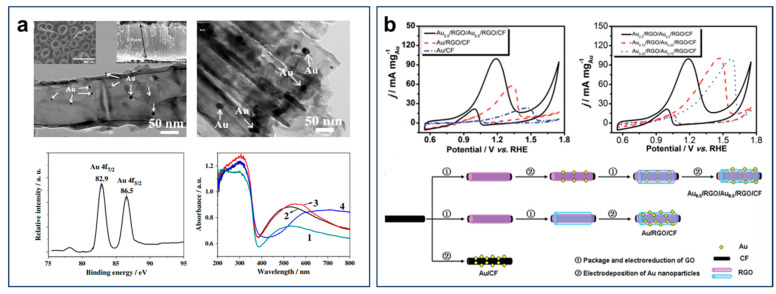
(**a**) TEM image, high-resolution Au 4*f* XPS, and UV-vis diffuse reflectance spectra of Au/TiO_2_ NTs. Reproduced with permission from Reference [95]. Copyright 2011, *Electrochemistry Communications*. (**b**) Cyclic voltammetry curves and diagram of the synthesis process of Au_0.5_/RGO/Au_0.5_/RGO/CF. Reproduced with permission from Reference [96]. Copyright 2015, *Journal of Materials Chemistry A*.

The rational design of support materials has emerged as another pivotal strategy to enhance catalytic performance of Au-based catalysts by optimizing the dispersion, electronic structure, and interfacial interactions of Au nanoparticles [91,92,93,94]. This strategy not only facilitates improved ethanol and intermediate adsorption, thereby enhancing mass activity, but also improves long-term stability by mitigating nanoparticle aggregation and CO poisoning. For instance, Au/TiO_2_ NTs were prepared by loading low-content Au nanoparticles on highly ordered TiO_2_ nanotube arrays [95]. Au/TiO_2_ NTs showed significantly enhanced EOR activity and poisoning resistance, attributed to the strong synergistic interactions between TiO_2_ and Au nanoparticles (Figure 16a). Du et al. developed a three-dimensional Au nanoparticle/reduced graphene oxide (RGO)/carbon fiber (CF) hybrid electrode (denoted as Au_0.5_/RGO/Au_0.5_/RGO/CF) by a layer-by-layer method (Figure 16b) [96]. The excellent electronic conductivity of the RGO sheets benefited electron transfer and the removal of the intermediate species by oxidation during EOR, which improved EOR performance. The high electrical conductivity and large specific surface area of RGO could facilitate rapid electron transfer and intermediate oxidation (e.g., CO_ads_), while the three-dimensional CF framework could enhance mass transport and prevent Au nanoparticle aggregation. The optimized Au_0.5_/RGO/Au_0.5_/RGO/CF electrode exhibited a more negative oxidation peak potential, suggesting a low overpotential due to reduced mass transfer resistance. It highlights the essential role of support engineering in improving the activity, stability, and overall practicality of Au-based catalysts for DEFC applications.

### 4.5. Non-Noble Metal Catalysts

Noble metal electrocatalysts are widely recognized for their high catalytic activity; however, their high cost and scarcity hinder commercial viability. Moreover, CO poisoning further limits efficiency of DEFCs. These challenges can be mitigated by partially or fully replacing noble metals with non-noble metal electrocatalysts, which offer a cost-effective and catalytically active alternative. Beyond their economic and environmental advantages, non-noble metal catalysts achieve enhanced catalytic performance through nanostructure design, doping and alloying, and interface engineering [97,98]. Collectively, these breakthroughs not only narrow the performance gap between non-noble metal catalysts and noble metals but also pave the way for developing high-efficiency, durable non-noble metal catalysts.

**Figure 17:**
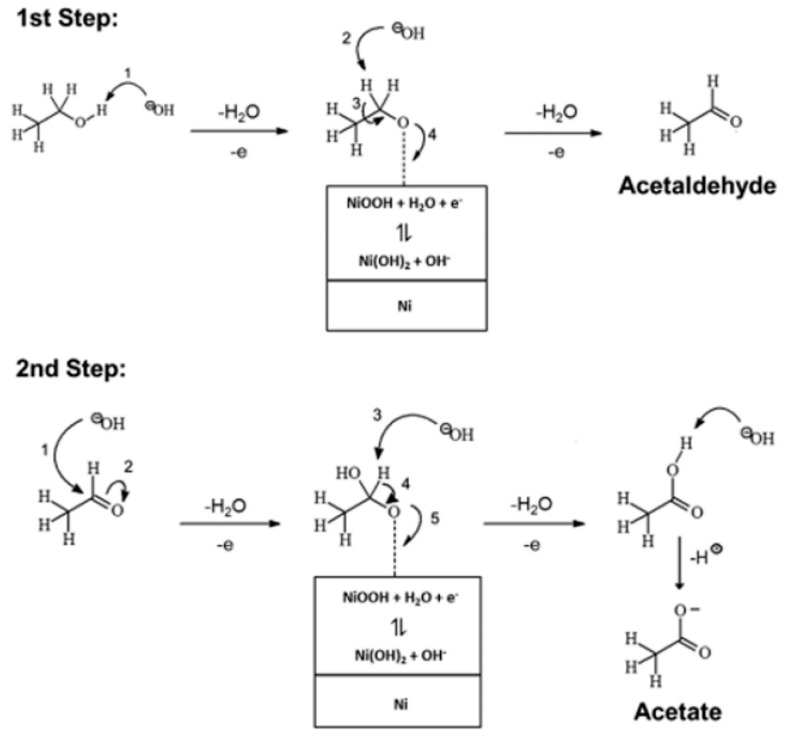
Mechanism of ethanol electrooxidation on nickel electrode. Reproduced with permission from Reference [95]. Copyright 2015, *Journal of Electroanalytical Chemistry*.

Extensive studies have demonstrated that Ni-based catalysts exhibit remarkable electrocatalytic activity toward the EOR in alkaline electrolytes [99,100,101]. The outstanding performance is primarily attributed to the formation of a highly active NiOOH intermediate during the catalytic process. As shown in Figure 17, Ni compounds are initially oxidized to generate NiOOH, a potent oxidizing species that facilitates ethanol into CH_3_COOH, which subsequently reacts with OH^−^ to form CH_3_COO^−^ [102]. NiOOH is reduced after oxidizing ethanol and is then re-oxidized under an applied potential, thereby establishing a self-catalytic cycle [103,104,105,106]. Consequently, under appropriate overpotentials, Ni-based catalysts not only achieve high catalytic activity but also attain a 100% selectivity for C2 products, as they neither adsorb ethanol nor induce C–C bond cleavage. Thus, the EOR performance of Ni-based catalysts is closely correlated with the ability to form NiOOH. The structural form of Ni-based catalysts—whether nanosheets, nanowires, or porous frameworks—directly influences the density of accessible NiOOH sites and mass transport, thus modulating catalytic performance. Transition metals such as Fe, Co, etc., also exhibit similar catalytic processes for the EOR, where the dynamic interconversion of multiple oxidation states and the generation of reactive oxygen species facilitate ethanol adsorption and activation [97,98,107]. These catalysts share similar reaction pathways and comparable distributions of intermediate products. However, non-precious metal catalysts commonly face challenges such as low intrinsic activity and the facile deactivation of active sites, which can be mitigated through strategic surface modifications and optimization of reaction conditions to enhance overall catalytic performance.

**Figure 18:**
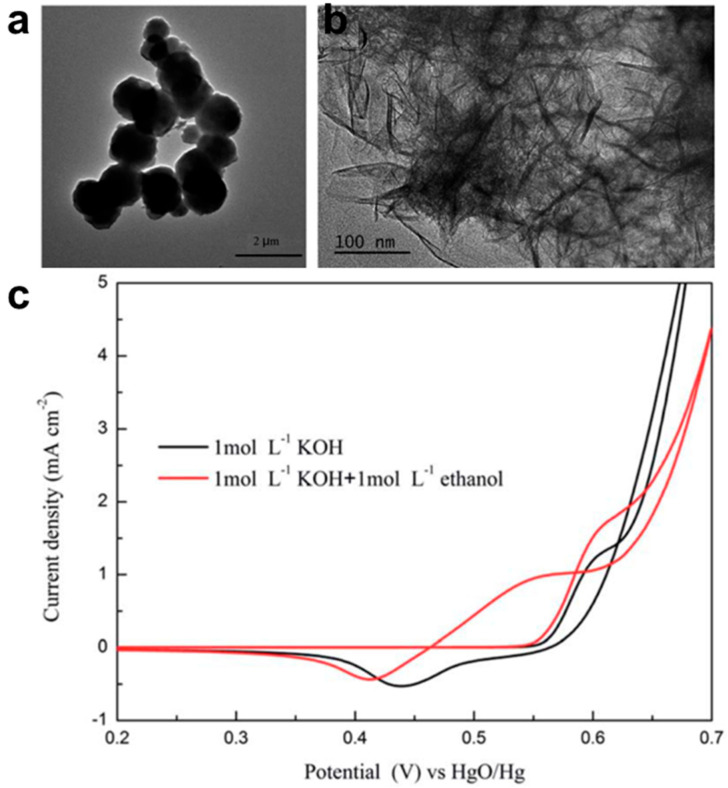
(**a**) TEM, (**b**) HRTEM, and (**c**) CVs of Ni–Fe LDH@MnO_2_. Reproduced with permission from Reference [108]. Copyright 2015, *RSC Advances*.

Building on the key role of the NiOOH in enhancing EOR performance, recent studies have focused on integrating other non-noble metal compounds into Ni-based catalysts. The incorporation of secondary transition metals (e.g., Fe, Co, Mn, or Cu) into Ni lattices has been shown to significantly alter the *d*-band center of Ni, thereby tuning its electronic structure. Such modulation optimizes the adsorption energy of OH^−^ and ethanol intermediates, enhancing both activity and tolerance against poisoning. Beyond composition, morphology also plays a decisive role. Core–shell architectures, heterojunctions, and defect-rich surfaces can significantly enhance activity by exposing high-index facets, introducing lattice strain, or facilitating charge transport. Defect sites often serve as preferential centers for OH^−^ adsorption, thereby accelerating the initial steps of ethanol oxidation. Wang et al. prepared manganese-dioxide-loaded Ni–Fe LDH nanospheres (Ni–Fe LDH@MnO_2_) using a simple liquid-phase synthesis method (Figure 18) [108]. Although MnO_2_ alone does not exhibit significant EOR activity, the LDH@MnO_2_ microspheres displayed enhanced EOR activity and stability compared to Ni–Fe LDH. The improvement was attributed to MnO_2_ promoting OH_ads_ adsorption on the Ni–Fe LDH surface, which facilitated the formation of the active NiOOH and FeOOH, lowered the self-oxidation potential, and regenerated active sites through reactions with adsorbed OH_ads_ species and intermediates. Furthermore, integrating metallic Ni with the perovskite LaMnO_3_ substantially altered the catalyst’s physicochemical properties and enhanced its EOR performance [106]. In this composite, Ni was dispersed within the LaMnO_3_ framework as NiO_x_ clusters, forming unique Ni–O–Mn active sites that are critical for improving both EOR activity and stability. Hamid et al. fabricated Ni–Cr_2_O_3_ composite materials with small particle sizes via electrodeposition and optimized their activity by tuning the Cr_2_O_3_ content [109]. Meng et al. doped Ni into m-CoSeO_3_, modifying the catalyst structure and enhancing its EOR performance [110]. Collectively, these studies highlight the potential of non-noble metal composites to overcome the intrinsic limitations of Ni-based catalysts and provide promising avenues for the design of high-performance EOR systems.

Non-metal doping could also effectively enhance its EOR performance [99,111,112,113]. Electronegative elements such as S, Se, and P induce charge redistribution at adjacent Ni sites, weakening Ni–Ni metallic bonding and creating electron-rich or electron-deficient centers. This modulation lowers the energy barrier for ethanol dehydrogenation. Zhuang et al. synthesized defective Ni_3_S_2_ nanowire catalysts with abundant surface defects, which achieved a current density of 106.4 mA cm^−2^ at 1.5 V vs. RHE with a C2 product selectivity of 99% [114]. Moreover, the incorporation of S into Ni compounds effectively modulates the electronic structure, thereby reducing the oxidation potential of Ni-based catalysts during the EOR. The current densities at 1.5 V (vs. RHE) were 32.8, 43.3, and 57.4 mA cm^−2^ for NiO, NiS, and NiSe in 1.0 M KOH with 1.0 M ethanol at a scan rate of 50 mV s^−1^. The acetic acid yields were 92%, 99%, and 97% for NiO, NiS, and NiSe, respectively. This indicated that all the three types of Ni chalcogenides showed high C2 selectivity [115]. Subsequent compositional tuning of NiSe catalysts demonstrated that increasing the Se content further enhanced EOR performance, with the activity order being NiSe_2_ > NiSe > Ni_3_Se_2_. This indicates that an increased Ni–Ni distance in Ni-based compounds facilitates the formation of the active NiOOH species during the EOR process, which reduces the dehydrogenation energy barrier and further improves EOR efficiency. Overall, it highlights that non-metal doping is a critical factor in tailoring the catalytic properties of non-noble-based materials for enhanced EOR performance.

Non-noble metal catalysts exhibit promising EOR activity at specific overpotentials; however, they predominantly drive the oxidation of ethanol to C2 products via a four-electron transfer mechanism and lack the ability to cleave the C–C bond to produce lower-carbon compounds [85]. As a result, ethanol undergoes only partial oxidation, yielding intermediates such as CH_3_CHO or CH_3_COOH instead of being fully oxidized to CO_2_, which limits both energy conversion efficiency and reaction selectivity. Furthermore, these catalysts suffer from poor chemical stability in strongly alkaline or acidic environments—they are prone to dissolution, corrosion, and structural reorganization—ultimately leading to rapid catalytic deactivation and shortened operational lifetimes.

### 4.6. Comparative Mechanistic Insights in EOR Catalysts

To provide a clearer benchmark for evaluating the catalytic performance of different EOR systems, we have summarized representative catalysts reported in the recent literature and compared them with the commercial Pt/C benchmark (Table 1).

Beyond summarizing prior studies, it is important to critically compare the relative merits of different catalyst systems (Table 2). The application prospects of Pt, Pd, Rh, Au, and non-noble metals for the EOR are largely determined by their catalytic mechanisms, selectivity, and resistance to poisoning. Pt exhibits high catalytic activity for the EOR but suffers from strong CO adsorption, leading to severe CO poisoning and catalytic performance degradation. In alkaline media, Pd shows strong C2 selectivity, predominantly producing CH_3_COOH. While Pd exhibits weaker CO adsorption compared to Pt, its inherent tendency for partial oxidation limits overall energy conversion efficiency, necessitating further modifications, such as alloying and surface engineering, to improve C1 selectivity. Au can serve as a stabilizer or promoter in bimetallic and nanostructured catalysts, enhancing EOR activity and selectivity. Rh is unique in its ability to efficiently cleave the C–C bond, favoring the complete oxidation pathway to CO_2_ with high C1 selectivity, making it one of the most promising catalysts for high-efficiency ethanol oxidation despite its high cost. Non-noble metals, including Ni, Co, and Fe, provide cost-effective alternatives but generally lack the ability to break C–C bonds. For instance, Ni-based catalysts follow a NiOOH-mediated oxidation mechanism in alkaline media, demonstrating moderate activity and stability. Their performance can be improved through compositional tuning, doping, and hybridization with transition metals or metal oxides. However, non-noble metals face significant challenges regarding chemical stability in highly acidic or alkaline environments. The selection of optimal catalysts (Pt, Pd, Rh, Au, or non-noble metals) for the EOR must strategically balance intrinsic activity, selectivity, poisoning resistance, and cost-effectiveness, being tailored to specific operational environments and energy conversion objectives.

## 5. Recent Advances in Electrocatalyst Design for EOR

Significant advancements have been achieved in the development of EOR catalysts; however, challenges such as limited catalytic performance, high cost, and inadequate long-term stability remain key barriers to large-scale application. Addressing these limitations requires the rational design of cost-effective and highly efficient catalytic systems. Notably, the advances in single-atom catalysts (SACs) have demonstrated the potential to enhance catalytic performance by precisely tuning electronic structures and optimizing active site utilization. Coupled with low-cost supports such as carbon-based materials and metal–organic frameworks (MOFs), these catalysts effectively reduce noble metal usage while improving stability and resistance to poisoning, thereby ensuring prolonged operational viability. To further enhance catalyst durability, extensive research has been devoted to surface modification, self-healing designs, and the integration of robust support materials. Functionalization strategies involving hydroxyl groups incorporation, nitrogen doping, and oxide coatings have proven effective in regulating adsorption behavior and mitigating catalyst deactivation. Additionally, structural engineering strategies, such as core–shell architectures, heterojunctions, and composite oxides, offer enhanced protection against sintering and degradation under operational conditions, thereby extending catalyst lifespan and improving practical applicability. The mechanistic understanding of the EOR has also benefited from advancement of in situ characterization techniques, such as X-ray absorption spectroscopy, neutron scattering, and in situ Raman spectroscopy, which offer real-time insights into reaction intermediates and active site dynamics. Computational approaches, including molecular dynamics simulations and density functional theory, have further elucidated reaction energetics and facilitated the rational design of EOR catalysts. Furthermore, the integration of machine learning and high-throughput screening methodologies is revolutionizing catalyst discovery by enabling the rapid prediction of structure–property relationships and accelerating the identification of optimal catalytic configurations.

Taken together, the collective advances in catalyst design, mechanistic elucidation, and system-level integration have established a solid foundation for future innovations in EOR electrocatalysis. Nevertheless, critical scientific and engineering challenges remain unresolved, necessitating forward-looking strategies and interdisciplinary collaboration. These aspects are discussed in the following outlook section.

## 6. Outlook and Future Perspectives

Despite considerable progress in the design and understanding of electrocatalysts for the EOR, several fundamental and practical challenges remain unresolved, limiting the widespread deployment of DEFCs. A critical barrier lies in the persistent trade-off between catalytic activity, selectivity, and durability, particularly under dynamic operational environments. Future breakthroughs will depend on the precise modulation of active site coordination environments and reaction pathways, which requires the integrated advancement of in situ/operando characterization techniques and predictive theoretical models. Employing a closed-loop research paradigm of rational design (design–fabrication–characterization–mechanism–redesign), integrated with multi-scale modeling and advanced in situ characterization, is crucial for accelerating the development of next-generation EOR electrocatalysts.

Beyond catalyst development, the performance of DEFCs is determined not only by catalyst innovation but also by system-level factors, including anode–cathode synergy, durable proton-conducting membranes, electrode microstructure, and low-resistance bipolar plates. A paradigm shift toward multi-scale optimization—integrating material-level breakthroughs with device- and stack-level engineering—is critical for enhancing energy conversion efficiency and facilitating the practical deployment of DEFCs in portable electronics, transportation, and high-energy-density power systems. The continued convergence of experimental, theoretical, and engineering studies is expected to drive significant progress in EOR catalyst development, bridging the gap between fundamental research and industrial implementation. With sustained efforts, DEFCs hold great promise as a viable clean energy solution, contributing to global sustainability goals and the transition toward a low-carbon energy future.

Sustainability considerations—such as minimizing noble metal usage, enabling scalable synthesis, and ensuring component recyclability—must be incorporated from the earliest stages of catalyst and system design. Simultaneously, addressing both the scalability and durability of EOR electrocatalysts is essential for translating laboratory breakthroughs into practical DEFC systems. Current synthesis of advanced nanostructures, including single-atom catalysts, heterostructures, and defect-rich materials, remains limited to small-scale, costly approaches. Future efforts should focus on reproducible, low-cost, and environmentally friendly techniques—such as electrodeposition, spray-coating, 3D printing, and continuous-flow synthesis—that preserve structural precision and catalytic performance at scale. Long-term durability under realistic operating conditions, challenged by sintering, corrosion, phase transformations, and dynamic interface restructuring, also requires rational strategies combining defect engineering, robust support integration, and surface modification to stabilize active sites and suppress degradation. Standardized accelerated stress tests and durability benchmarks will further enable fair comparisons across catalyst classes and with commercial references. By concurrently addressing sustainability, scalability, and durability, future research can accelerate the development of high-performance catalysts and advance the real-world implementation of DEFC technology.

In summary, the road ahead for EOR electrocatalysis and DEFC technology is both challenging and full of opportunities. By harnessing the convergence of emerging characterization tools, intelligent design frameworks, and scalable engineering strategies, the field is poised to deliver practical, efficient, and environmentally sustainable energy solutions for clean power technologies.

## Figures and Tables

**Table 1:** A comprehensive summary of representative EOR catalysts.

Catalyst Composition	Electrolyte	Activity	Product Selectivity	Stability Test	References
Pt/C	0.1 M HClO_4_ + 0.5 M EtOH	260 mA·mg^−1^_metal_	main C2	190 mA·mg^−1^ (after 500 CVs)	[25]
Pt_1_Rh_1_ ANDs	1.0 M KOH + 1.0 M EtOH	462.1 mA·mg^−1^_metal_	main C2	400.6 mA·mg^−1^ (after 1000 CVs)	[24]
Pt_74_Mn_21_Ir_5_	0.1 M HClO_4_ + 0.5 M EtOH	1020 mA·mg^−1^_metal_	main C2	870 mA·mg^−1^ (after 500 CVs)	[25]
Pt_59_Cu_41_	0.5 M KOH + 0.5 M EtOH	5580 mA·mg^−1^_metal_	main C2	2.44 mA cm^−2^ (after 3600 s)	[27]
4H-Au@4H-PtCu	1.0 M KOH + 1.0 M EtOH	4220 mA·mg^−1^_metal_	main C2	~20 mA·mg^−1^ (at 0.7 V, after 3000 s)	[34]
PtZn	1.0 M KOH + 2.0 M EtOH	1029.7 mA·mg^−1^_metal_	main C2	~7.5 mA·mg^−1^ (at 0.7 V, after 2 h)	[35]
PtBi@Pt	1.0 M KOH + 1.0 M EtOH	9010 mA·mg^−1^_metal_	main C2	7080 mA·mg^−1^ (after 5000 CVs)	[36]
PtCu/Cu_2− x_Se	1.0 M KOH + 1.0 M EtOH	5030 mA·mg^−1^_metal_	main C2	250 mA·mg^−1^ (at 0.7 V, after 2 h)	[38]
PtSn	0.2 M H_2_SO_4_ + 0.2 M EtOH	673.6 mA·mg^−1^_metal_	main C2	497.8 mA·mg^−1^ (after 5000 CVs)	[41]
Cu_1_Au_0.15_Pd_1.5_	0.5 M KOH + 0.5 M EtOH	1250 mA·mg^−1^_metal_	main C2	812.5 mA·mg^−1^ (after 8000 CVs)	[55]
PdCo NTAs	1.0 M KOH + 1.0 M EtOH	1562.1 mA·mg^−1^_metal_	main C2	200 mA·mg^−1^ (after 550 s)	[58]
PdMn-N_4_	1.0 M NaOH + 2.0 M EtOH	3740 mA·mg^−1^_metal_	main C2	3553 mA·mg^−1^ (after 8000 CVs)	[59]
PdCu	1.0 M NaOH + 1.0 M EtOH	10,590 mA·mg^−1^_metal_	main C2	7070 mA·mg^−1^ (after 250 CVs)	[60]
NiO-PdNi	1.0 M KOH + 1.0 M EtOH	1201.5 mA·mg^−1^_metal_	main C2	0.045 mA·mg^−1^ (after 30,000 s)	[61]
Pd/S&F–C	1.0 M KOH + 1.0 M EtOH	22,000 mA·mg^−1^_metal_	main C2	19,800 mA·mg^−1^ (after 10,000 CVs)	[62]
a-PdP_0.1_	1.0 M KOH + 1.0 M EtOH	4851 mA·mg^−1^_metal_	main C2	220 mA·mg^−1^ (after 3600 s)	[63]
Pd–Ni–P	1.0 M NaOH + 1.0 M EtOH	4950 mA·mg^−1^_metal_	main C2	215.4 mA·mg^−1^ (after 2000 s)	[64]
CPT Rh NBs	1.0 M NaOH + 1.0 M EtOH	185.3 mA·mg^−1^_metal_	14.5% C1	122.3 mA·mg^−1^ (after 1200 s)	[70]
Rh nanobranches	1.0 M NaOH + 1.0 M EtOH	79.1 mA·mg^−1^_metal_	15.8% C1	/	[71]
Pb@Rh	1.0 M NaOH + 1.0 M EtOH	1454 mA·mg^−1^_metal_	21% C1	828.8 mAmg^−1^ (after 20,000 s)	[74]
Rh_85_Ni_15_	1.0 M NaOH + 1.0 M EtOH	159 mA·mg^−1^_metal_	16% C1	~0.7 mA cm^−2^ (after 1000 s)	[75]
Rh–Pb	1.0 M NaOH + 1.0 M EtOH	2636 mA·mg^−1^_metal_	20% C1	57% retention (after 10,000 s)	[78]
Rh-Bi(OH)_3_	1.0 M NaOH + 1.0 M EtOH	3500 mA·mg^−1^_metal_	26.2% C1	25% retention (after 4 h)	[79]
PdRh NBs	1.0 M KOH + 1.0 M EtOH	682 mA·mg^−1^_metal_	main C2	/	[82]
nanoporous Au	1.0 M KOH + 1.0 M EtOH	308 mA·mg^−1^_metal_	main C2	268.3 mA·mg^−1^ (after 800 CVs)	[87]
Au/C	0.1 M KOH + 1.0 M EtOH	140 mA·mg^−1^_metal_	main C2	65.0 mA·mg^−1^ (after 2000 s)	[87]
Pd@Au	1.0 M KOH + 1.0 M EtOH	~800 mA·mg^−1^_metal_	main C2	2 mA (after 3600 s)	[88]
Au_0.5_/RGO/Au_0.5_	1.0 M KOH + 1.0 M EtOH	100.5 mA·cm^−2^	main C2	~0.4 mA·mg^−1^ (after 4000 s)	[96]
α-Ni(OH)_2_	1.0 M KOH + 1.0 M EtOH	40 mA·cm^−2^	main C2	/	[103]
Ni HS	1.0 M KOH + 1.0 M EtOH	17 mA·cm^−2^	main C2	/	[104]
Ni–Fe LDH@MnO_2_	1.0 M KOH + 1.0 M EtOH	~5 mA·cm^−2^	main C2	/	[108]
Ni-Cr_2_O_3_	1.0 M NaOH + 2.0 M EtOH	~300 mA·cm^−2^	main C2	/	[109]
Ni-6/CX/G	1.0 M NaOH + 2.0 M EtOH	5925 mA·cm^−2^	main C2	/	[113]
Ni_3_S_2_ NWs	1.0 M KOH + 1.0 M EtOH	106 mA·cm^−2^	main C2	/	[114]
NiSe_2_ NWs	1.0 M KOH + 1.0 M EtOH	70.2 mA·cm^−2^	main C2	/	[115]

**Table 2:** Comparative strengths and weaknesses of representative EOR catalysts.

Catalyst System	Strengths	Weaknesses
Pt	Benchmark activity in acidic media Strong ability to activate ethanol and intermediates	Severe CO poisoning Low CO_2_ selectivity
Pd	High intrinsic activity in alkaline media Favorable adsorption of ethanol and intermediates	Low CO_2_ selectivity Surface easily oxidized and deactivated
Rh	Strong ability to cleave C–C bond Relatively low initial potential	Extremely high cost and scarcity Lower overall activity compared to Pt/Pd
Au	Excellent resistance to CO poisoning Stable under alkaline conditions	Low CO_2_ selectivity Poor activity in acidic media
Ni, Co, etc.	Abundant and low-cost High activity in alkaline media	Low CO_2_ selectivity Surface easily oxidized and corroded Limited activity in acidic electrolytes
